# Editorial: Management of pancreatic cancer: Defining the targets for therapy

**DOI:** 10.3389/fmed.2022.971067

**Published:** 2022-08-03

**Authors:** Nam Q. Nguyen

**Affiliations:** ^1^Department of Gastroenterology and Hepatology, Royal Adelaide Hospital, Adelaide, SA, Australia; ^2^School of Medicine, University of Adelaide, Adelaide, SA, Australia

**Keywords:** pancreatic cancer, targeted therapy, biomarkers, patient-derived-organoid (PDO), CHFR, high mobility group box 1 (HMGB1)

Despite advances in diagnosis and management, pancreatic ductal adenocarcinoma (PDAC) is still lethal with a 5-year survival rate varies between 9 and 11% (1, 2). More importantly, the incidence of pancreatic cancer is increasing and is projected to be the second most cause of cancer-related death by 2030 (1), prompting a need for high clinical impact research in this cancer. The combination of aggressive tumor behavior, late presentation and poor response to chemotherapy are responsible for its poor outcomes, with only 20% of patients deemed “resectable” at the time of diagnosis. Even then, the rate of cancer recurrence at the resection site or distance metastases is high (up to 80%), with a 5-year survival rate of only 20–30%. One reason for such disappointing results is the occurrence of occult metastasis in the early stage of the disease, too small to be detected by any form of imaging, including endoscopic ultrasound, MRI and PET scans. This is best shown by the presence of circulating tumor cells in the portal vein of all patients who are deemed to have “resectable” cancer (3), reflecting the aggressive tumor behavior of pancreatic cancer. In fact, whole genome evaluation of 100 pancreatic cancers confirmed that it is a highly complex cancer with over 5,000 mutations (4), multiple oncogenic pathways (5) and chromosomal anomalies (6). With these insights, it is more logical to characterize and prognosticate the cancer based on its tumor behaviors, which can clinically reflect by biomarkers, or more recently, a panel of mutations using next-generation sequencing (NGS) or whole genome sequencing (WGS) (4–6). Understanding the role of these biomarkers and mutations is key to facilitate early-stage diagnosis, prognosticate and guide treatment in pancreatic cancer. The combination of accurate imaging techniques and identification of novel biomarkers, therefore, is crucial to improve prognostication and treatment outcomes of PDAC. In this issue, two of the potential novel biomarkers and models for developing targeted treatments of PDAC are discussed.

Immune checkpoints have been increasingly used as target for precision oncological treatment for many cancers, including PDAC. Checkpoint with forkhead-associated and ring finger domains (CHFR) gene encodes a protein implicated in mitosis entry checkpoint. Although CHFR has been explored in various cancers, data on its role in PDAC remains limited. In the first article of this issue, González-Borja et al. prospectively examine the impacts of CHFR expression and promoter methylation on the outcome of patients with borderline and resectable PDAC. In both groups, the progression free survival was significantly longer in patients with stronger intensity CHFR expression (12.74 months vs. not reached; *p* = 0.025). Lower methylation levels were associated with longer overall survival (HR = 0.32; *p* = 0.042). Up to date, there are 4 studies that have explore the role of CHFR in PDAC, with conflicting data (7–9). Increased CHFR promoter methylation was found to be associated with higher lymph node metastasis, supporting Borja's findings (7). Based on immunohistochemical expression of CHFR, high level expressions were associated with decreased proliferative rate, early T-stage disease and improved prognosis in patients with resectable PDAC (8, 9). The only study that did not show any correlation between CHFR expression and overall survival was by Wei et al., in which the study sample size was small (*n* = 27) and adjuvant chemotherapy was with docetaxel rather than FOLFIRINOX (9). Although available data, including those from González-Borja et al., support the use of CHFR expression or its methylation as a prognostic marker in patients with resectable PDAC, the evaluation of its prognostic role should be extended to non-resectable PDAC, which account for 80% of all PDAC. In addition, future research should focus on the correlation of CHFR expression with responses to chemotherapeutic agents in PDAC, allowing identification of potential targeted therapies for CHFR-expressed PDACs.

Tumor suppressors are well-known oncological regulators, and in this issue, the role of high mobility group box 1 (HMGB1), a protein involved in inflammation and intracellularly suppresses oncogenic pathways, in response to radiation therapy, was assessed in PDAC (10). In pancreatic cancer mouse models, the impact of HMGB1 expression on tumorigenesis depends on its cellular location. The intra-cellular levels of HMGB1 regulate its growth, with depleted levels accelerating K-Ras-driven carcinogenesis (10, 11). However, the effects of HMGB1 is reversed once the protein is outside of the cells, with high extracellular HMGB1 levels found to stimulate pancreatic cancer cell growth (12). Using pancreatic cancer cell lines (PANC-1 and SW1990), Zhu et al. explored the impact of radiotherapy on the supernatant HMGB1 levels. Compared to control (no radiation exposure), a 4Gy (PANC-1) to 10Gy (SW1990) exposure increased release of HMGB1 into the extracellular space, resulting in significantly higher extracellular HMGB1 levels in irradiated cell lines. These findings are similar to those from 2 previous studies, demonstrating that HMGB1 is released from apoptotic cells after irradiation (13, 14), and the elevated extra-cellular HMGB1 levels were associated with higher proliferation rate of pancreatic cancer (61.4 vs. 45.9% in SW1990 cell line, *p* < 0.05; 47.5 vs. 38.4% in PANC-1 cell line, *p* < 0.05). More importantly, when a HMGB1 inhibitor was added, proliferation rates fell to near control levels. In humans, high serum HMGB1 levels have been shown to associated with advanced-stage PDAC (15). These results are highly relevant as they may explain the discrepancies in the benefits of radiation therapy in PDAC over the last 3–4 decades. In fact, a RCT has shown that combined broad-beam radiotherapy with chemotherapy are harmful for PDAC (11). The survival benefits of radiation therapy in PDAC have only been found with *focused high dose* (40 to 50Gy) radiation in the form of stereo-static body radiation therapy (SBRT) (16). It is a priority for more research to look at the impact of high dose radiation on extra-cellular HMGB1 levels in PDAC, as well as potential therapeutic targets to inhibit extracellular HMGB1 in the management of PDAC.

Even with the tremendous efforts over the last decade to define the genomic anomalies and appropriate biomarkers or mutations to guide chemotherapy, only a small number of patients with PDAC have been found to have actionable mutations (26–30%) in clinical practice. The real-life success of biomarker driven chemotherapy, therefore, has been very limited (17). The alternative approach is generation of patient-derived preclinical cancer models to identify effective treatments. Patient-derived xenografts allows for vascularisation of the engrafted cell/tissue with cells resembling true tumor structure and can reveal a response specific to the patient. However, xenograft models are time consuming (up to 6 months to grow), costly, and involves many animals (18). Pancreatic cancer patient-derived-organoids (PDOs), as described by PIro et al., can overcome the weaknesses of xenografts, being cheaper, having a shorter generation duration (2–4 weeks), and does not require animal experimentation. More importantly, the generated organoids can retain the original pancreatic cancer genomic signatures and heterogeneity, simulating patient-specific cancer and allowing reliable drug-specific testing. Thus far, most studies reported on PDOs are typically developed from resected human pancreatic tumors, which poses a significant limitation in that less than 20% patients with PDAC are resectable. EUS-guided sampling has provided a less invasive approach and allows organoids to be developed in all patients with PDAC. Recent data indicated that an EUS-guided approach can be feasible in up to 87% of cases (19). To have this concept integrated into routine clinical care, further studies are needed to standardize protocols for EUS-guided sampling and techniques of PDO generation and identify an appropriate panel of chemotherapeutic agents.

Given EUS core biopsy can provide adequate tissue for both biomarker/genomics analysis and generating PDO for drug testing, it likely that both techniques will be utilized in parallel to optimize the management of PDAC in the near future. The ability to characterize the cancer biology rapidly by either WGS or NGS will not only allow the physician to prognosticate the disease but also identify any targeted therapy. However, if there is no “actionable” mutation found, drug testing from PDO can help to identify the most effective chemotherapy.

## Author contributions

NN formed the concept of the article and contributed to the writing and editing of the final draft. The author confirms being the sole contributor of this work and has approved it for publication.

## Conflict of interest

The author declares that the research was conducted in the absence of any commercial or financial relationships that could be construed as a potential conflict of interest.

## Publisher's note

All claims expressed in this article are solely those of the authors and do not necessarily represent those of their affiliated organizations, or those of the publisher, the editors and the reviewers. Any product that may be evaluated in this article, or claim that may be made by its manufacturer, is not guaranteed or endorsed by the publisher.
